# Ultraselective transcatheter arterial embolization with small-sized microcoils for acute lower gastrointestinal bleeding

**DOI:** 10.1186/s42155-021-00215-9

**Published:** 2021-03-09

**Authors:** Mitsuhiro Kinoshita, Hiroshi Kondo, Suguru Hitomi, Takuya Hara, Ryusei Zako, Masayoshi Yamamoto, Junichiro Hiraoka, Yukiko Takaoka, Hideaki Enomoto, Naoki Matsunaga, Katsuya Takechi, Ryozo Shirono, Yoko Akagawa, Kyosuke Osaki, Norio Ohnishi, Hayato Tani

**Affiliations:** 1 Department of Radiology, Tokushima Red Cross Hospital, 103 Irinokuchi Komatsushima-cho, Komatsushima City, Tokushima 773-8502 Japan; 2 Department of Radiology, Teikyo University School of Medicine, 2-11-1 Kaga Itabashi-ku, Tokyo, 173-8606 Japan; 3 Department of Radiology (Diagnostic Radiology), Tokushima University Hospital, 2-50-1, Kuramoto-cho, Tokushima City, Tokushima 770-8503 Japan; 4 Department of Emergency & Clinical Care Medicine, Tokushima Red Cross Hospital, 103 Irinokuchi Komatsushima-cho, Komatsushima City, Tokushima 773-8502 Japan; 5 Department of Radiology, Kawashima-kai Kawashima Hospital, 1-39, Kitasakoichiban-cho, Tokushima City, Tokushima 770-0011 Japan

**Keywords:** Acute lower gastrointestinal bleeding, Microcoils, Recurrent bleeding, Ultraselective transcatheter arterial embolization, Vasa recta

## Abstract

**Purpose:**

To evaluate the clinical outcome of ultraselective transcatheter arterial embolization (TAE) with small-sized microcoils for acute lower gastrointestinal bleeding (LGIB).

**Materials and methods:**

The subjects were 17 consecutive patients (mean age, 69 years) with LGIB who were treated with ultraselective TAE using small-sized microcoils between December 2013 and December 2019. Ultraselective TAE was defined as embolization of one or both of the long or short branches of the vasa recta. The etiologies of bleeding were colonic diverticulosis in 16 patients (94%) and malignancy in one patient (6%). The bleeding foci were in the ascending colon in 11 patients (65%), transverse colon in 2 patients (12%), and sigmoid colon in 4 patients (23%). A total of 18 branches (diameter: range 0.5–1.5 mm, mean 1.1 mm) of the vasa recta in 17 patients were embolized with small-sized microcoils (size range 1–3 mm, mean combined lengths of all microcoils 7.6 cm). The mean follow-up period was 19 months (range 1–80 months). The technical and clinical success rate, recurrent bleeding rate, major complications and long-term clinical outcomes were retrospectively evaluated.

**Results:**

Technical and clinical success was achieved in all patients (17/17). The rates of early recurrent bleeding (within 30 days of TAE) and major complications were 0% (0/17). Recurrent bleeding occurred in one patient at 2 months after TAE, but was stopped with conservative treatment. There were no other bleeding episodes or complications in the follow-up period.

**Conclusion:**

Ultraselective TAE with small-sized microcoils is a highly effective and safe treatment modality for LGIB.

## Introduction

Lower gastrointestinal bleeding (LGIB) is defined as bleeding below the Treitz ligament, and includes jejunal, ileal, colonic and rectal bleeding. Transcatheter arterial embolization (TAE) has gained widespread acceptance as first-line treatment for acute LGIB in cases in which an endoscopic approach is not possible or ineffective (Barnert and Messmann 2009; Hur et al. 2014; Kim et al. 2017; Kwon et al. 2019; Tan et al. 2010). Efficacy rates for TAE in initial hemostasis in such cases have been reported to be over 85% (Hur et al. 2014; Kim et al. 2017; Kwon et al. 2019; Tan et al. 2010). TAE for LGIB is often performed with N-butyl cyanoacrylate (NBCA) and coils. These are common embolic agents that are used alone or in combination with other agents, and the safety and efficacy of TAE with NBCA or microcoils for treatment of LGIB have been shown (d'Othée et al. 2006; Funaki et al. 2001; Huang et al. 2011; Hur et al. 2014; Kim et al. 2017; Kodani et al. 2016; Koganemaru et al. 2012; Kuo et al. 2003; Kwon et al. 2019; Park et al. 2020; Shimohira et al. 2015; Tan et al. 2010; Teng et al. 2013; Yata et al. 2013). Superselection and embolization of the bleeding branch is ideal; however, several reports have found a higher recurrent bleeding rate with microcoils than in NBCA embolization because of recanalization or collateral circulation due to proximal embolization (Hur et al. 2014; Kim et al. 2017; Kwon et al. 2019; Vaidya et al. 2008).

Ultraselective TAE with a microcatheter advanced as close as possible to the bleeding site has been described in a few recent case reports (Koganemaru et al. 2012). However, the clinical outcomes of ultraselective TAE with small-sized microcoils for LGIB have not been examined in long-term follow-up of a large patient cohort. The purpose of this study was to evaluate the outcomes of this form of ultraselective TAE in patients with acute LGIB.

## Materials and methods

### Patients

The subjects were 19 consecutive patients with LGIB who were treated with ultraselective TAE using small-sized microcoils after unsuccessful endoscopic hemostasis at two hospitals between December 2013 and March 2020. All patients were hemodynamically stable. Seven patients were treated at Tokushima Red Cross Hospital (Tokushima, Japan) and 12 were treated at Teikyo University Hospital (Tokyo, Japan). Two patients were excluded because they underwent surgical resection after ultraselective TAE. Both had a history of multiple bleeding episodes for which they had been treated endoscopically. Therefore, surgical resection was performed when their condition stabilized after TAE because the attending physician feared rebleeding. Thus, 17 patients (15 men and 2 women; age range, 52–89 years; mean age, 69 years) were included in the study. The institutional review board approved this retrospective study, and no patient consent was required. All patients were informed about the benefits and potential risks of the procedure, and all provided written informed consent.

### Embolization technique

**Fig. 1:**
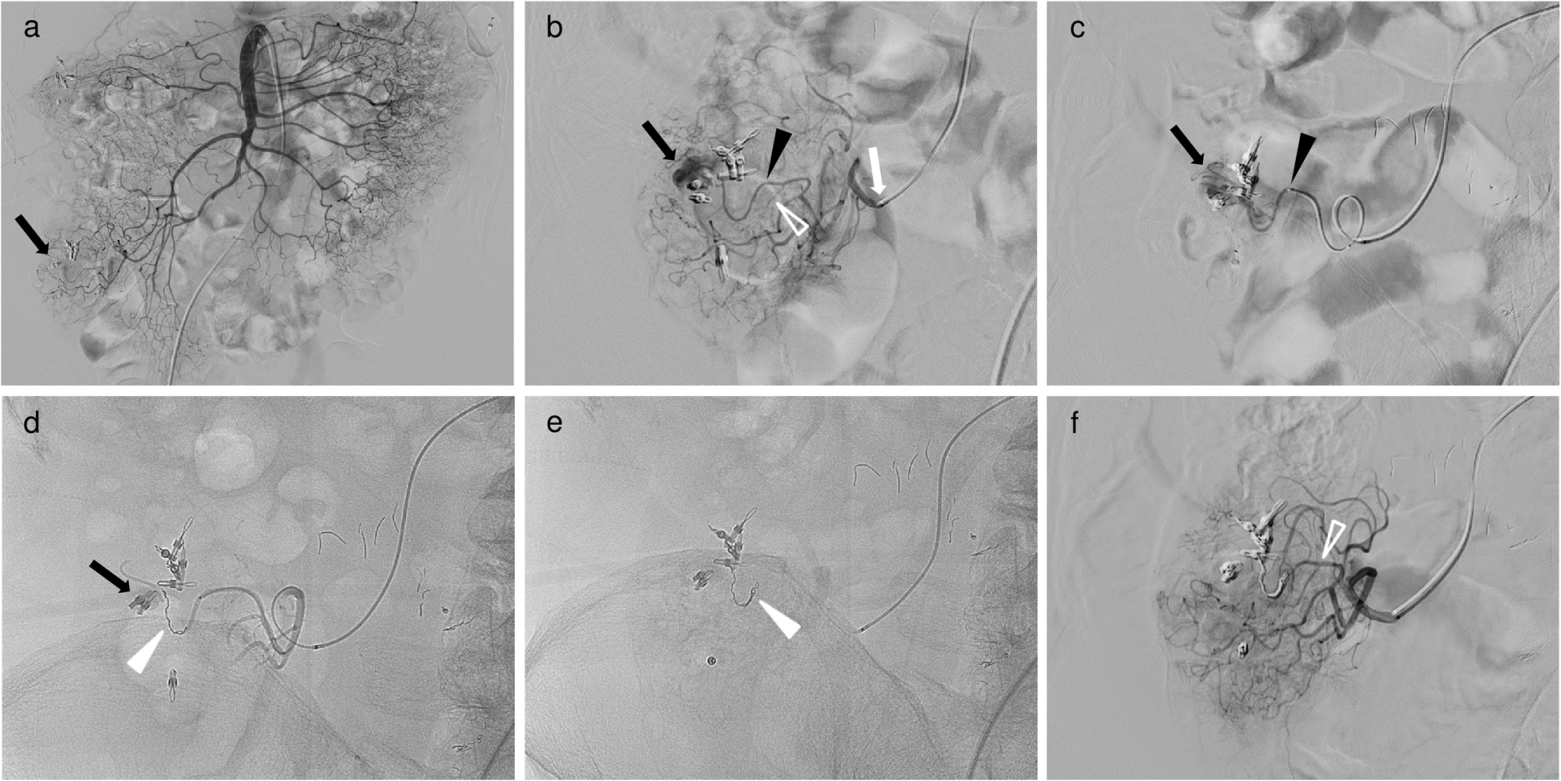
A 72-year-old man with ascending colonic hemorrhage due to colonic diverticulosis. Ultraselective TAE was performed with a triaxial system after hemostatic clipping via colonoscopy was unsuccessful. **a** Superior mesenteric angiography showed the vasa recta with near clipping (black arrow); however, contrast extravasation could not be identified. **b** Selective angiography through the colic branch (white arrow) of the ileocolic artery with a 2.6-F microcatheter (Masters HF; Asahi Intecc) showed a small and considerably bent vasa recta (long branch, black arrowhead; short branch, open white arrowhead) and a long branch of the vasa recta with contrast extravasation (black arrow). **c** Ultraselective angiography through the long branch of the vasa recta with a 1.6-F microcatheter (Carnelian Marvel S; Tokai Medical Products) (black arrowhead) showed contrast extravasation (black arrow). After identifying the bleeding site, a 1.6-F microcatheter was inserted into the bleeding branch as close as possible to the bleeding site (not shown). **d** Digital radiography showing a microcoil (Galaxy G3 MINI Microcoil; Codman & Shurtleff. Inc.) of 1 mm in diameter with a 2 cm coil (white arrowhead) placed at the site of contrast extravasation. However, contrast extravasation did not completely disappear (black arrow). **e** Digital radiography showing an additional microcoil (Galaxy G3 MINI Microcoil) of 1 mm in diameter with a 2 cm coil (white arrowhead) placed at the bleeding branch. **f** After ultraselective TAE, selective angiography through the colic branch showed no further contrast extravasation, occlusion of the long branch and maintenance of the short branch (open white arrowhead)

All patients underwent diagnostic computed tomography angiography (CTA) before TAE, and TAE was performed with reference to CTA. All except two TAE procedures were performed via the femoral artery using an angiographic system equipped with a flat-panel detector (Allura Clarity FD 20/15, Allura Xper FD 20/10, both Philips Healthcare, Best, The Netherlands; ACT FP 4100, INNOVA 4100, both GE Healthcare, Waukesha, WI, USA). Two procedures were performed via the left brachial artery due to bilateral common iliac artery occlusions or patient refusal of a femoral artery approach. First, diagnostic angiography was performed under local anesthesia to identify the bleeding site. A 4- or 5-F angiographic catheter (TWIST-B, SHA BOUDAI 2, both Medikit, Tokyo, Japan; Angiomaster, Heartcathe, both Terumo, Tokyo, Japan) was placed in the superior mesenteric artery (SMA) or inferior mesenteric artery (IMA) through a 4- or 5-F vascular sheath introducer (Medikit Catheter Introducer, Medikit; Radiforcus Introducer IIH, Terumo; Prelude Ideal, Merit Medical Systems, Inc., Salt Lake City, UT, USA). If the bleeding site could not be identified with SMA or IMA arteriography, arteriography from SMA or IMA branches (right colic, middle colic, ileocolic and sigmoid arteries) was performed using a 1.7- to 2.6-F microcatheter (Veloute, Masters HF, both Asahi Intecc, Aichi, Japan; Progreat λ17, Terumo; Peak Hunter, JMS, Tokyo, Japan). After identifying the bleeding site, ultraselective catheterization was performed using 1.6- or 1.7-F microcatheter (Veloute; Progreat λ17; Peak hunter; Carnelian Marvel S, Tokai Medical Products, Aichi, Japan) over a 0.014- or 0.016-in. guidewire (ASAHI Meister, ASAHI Meister S14, ASAHI CHIKAI V, all Asahi Intecc). With use of a 2.6-F high-flow microcatheter (Masters HF), we advanced a 1.6-F microcatheter (Carnelian Marvel S) into target arteries through a 2.6-F high-flow microcatheter (triaxial system) (Fig. 1) (Shimohira et al. 2015) because the marginal arteries were quite tortuous.

**Fig. 2:**
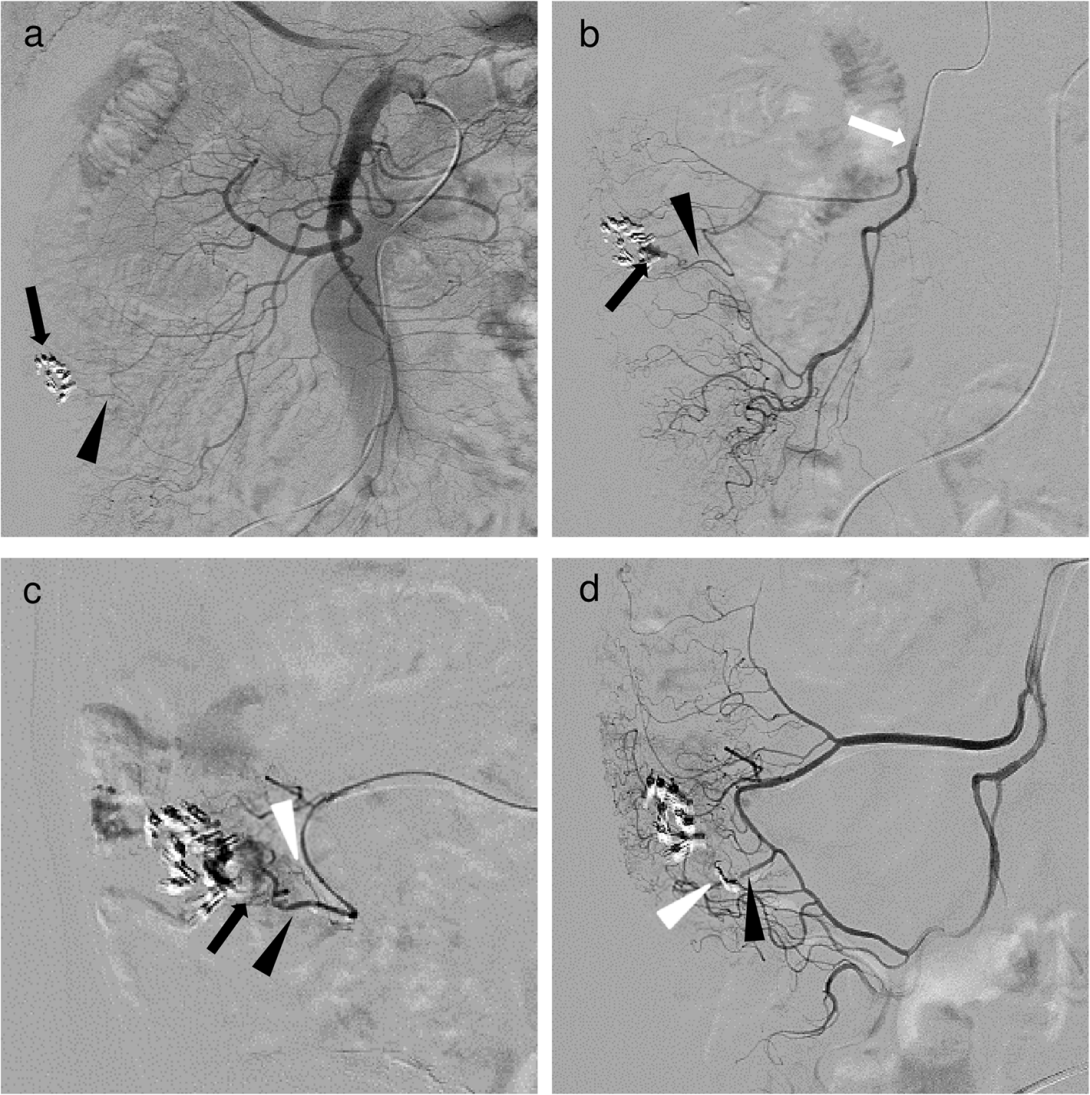
A 58-year-old man with ascending colonic hemorrhage due to colonic diverticulosis. Ultraselective TAE was performed after hemostatic clipping via colonoscopy was unsuccessful. **a** Superior mesenteric angiography image showed the vasa recta (black arrowhead) with near clipping (black arrow); however, contrast extravasation could not be identified. **b** Angiography through the ileocolic artery (white arrow) with a 1.7-F microcatheter (Veloute; Asahi Intecc) showed a small and considerably bent vasa recta (black arrowhead) and contrast extravasation (black arrow). After identifying the bleeding site, a 1.7-F microcatheter was inserted into the bleeding vasa recta (not shown). **c** Selective angiography through the vasa recta showed a short branch (white arrowhead) and contrast extravasation (black arrow) from the long branch (black arrowhead). **d** Two microcoils (Target Nano Coils; Stryker) of 1 mm in diameter and 2 cm long (white arrowhead) placed at the site of contrast extravasation and the bleeding branch. After ultraselective TAE, angiography through the ileocolic artery showed disappearance of extravasations, occlusion of the long branch and maintenance of the short branch (black arrowhead)

**Fig. 3:**
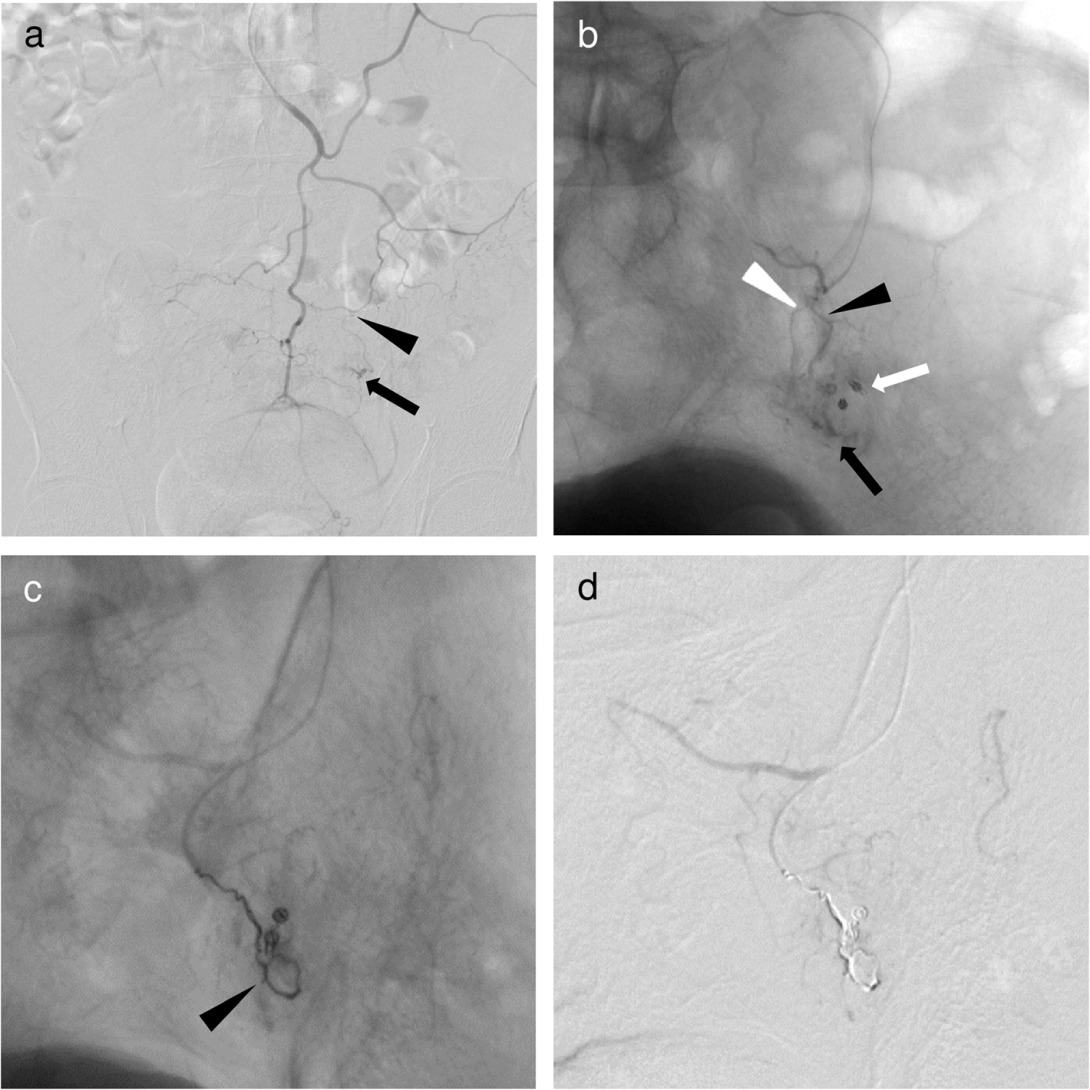
A 63-year-old man with sigmoid colonic hemorrhage due to colon cancer. Ultraselective TAE was performed after hemostatic clipping via colonoscopy was unsuccessful. **a** Inferior mesenteric angiography showed a small and considerably bent vasa recta (black arrowhead) of the sigmoid artery with contrast extravasation (black arrow) at the distal end. **b** Superselective angiography through the vasa recta (long branch, black arrowhead; short branch, white arrowhead) of the sigmoid artery with a 1.7-F microcatheter (Progreat λ17; Terumo) showed contrast extravasation (black arrow) at the distal end of near clipping (white arrow). After identifying the bleeding site, a 1.7-F microcatheter was inserted into the long branch of the vasa recta as close as possible to the bleeding site (not shown). **c** Ultraselective angiography through the long branch of the vasa recta showing a microcoil (Galaxy G3 Microcoil; Codman & Shurtleff. Inc.) of 3 mm in diameter and 8 cm long, and two microcoils of 2 mm in diameter and 2 cm long (black arrowhead) placed at the site of contrast extravasation and the bleeding branch. **d** After ultraselective TAE, contrast extravasation had completely disappeared

Ultraselective TAE was defined as embolization of one or both of the long or short branches of the vasa recta. A workstation (Interventional Workspot, Interventional Tools, both Philips Healthcare; AW 4.4, GE Healthcare) included in the angiographic system was used to determine artery diameters. The microcatheter was inserted into the long or short branches of the vasa recta as close as possible to the bleeding site and microcoils (Orbit Galaxy G3 Microcoil, Galaxy G3 MINI Microcoil, both Codman & Shurtleff. Inc., Raynham, MA, USA; Target Nano Coils, Target Ultra Coils, both Stryker, Fremont, CA, USA; Smart Coil, Penumbra, Inc., Alameda, CA, USA) ranging in size from 1 to 3 mm × 10 to 80 mm were deployed. Embolization was performed until no further arterial extravasation or pseudoaneurysm was seen (Figs. 1, 2 and 3).

### Assessment

Electronic medical records (EMRs) of patients and images of the procedures were reviewed, and a telephone interview was conducted. The technical and clinical success rates, early recurrent bleeding rate, major complications, and long-term clinical outcomes were retrospectively evaluated. Technical success was defined as disappearance of arterial contrast extravasation or pseudoaneurysm on angiography after embolization with target vessels filled with microcoils. Clinical success was defined as cessation of bleeding without further transcatheter, endoscopic or surgical interventions. Early recurrent bleeding was defined as rebleeding from the treated area after TAE within 30 days. Complications were evaluated based on the Cardiovascular and Interventional Radiological Society of Europe classification system for complications from grade 1 to grade 6 (Filippiadis et al. 2017). We defined complications of grade 3 (additional postprocedural therapy or prolonged hospital stay (> 48 h) required; no postprocedural sequelae), grade 4 (complication causing a permanent mild sequelae, resuming work and independent living), grade 5 (complication causing a permanent severe sequelae, requiring ongoing assistance in daily life), and grade 6 (death) as major complications. All other complications were considered to be minor. For long-term clinical outcomes, EMRs at our hospitals or at those to which a patient moved were reviewed for major complications and recurrent bleeding during follow-up for 14 patients. A telephone interview was conducted for 3 patients who were lost to follow-up visits.

## Results

The background of the patients is shown in Table 1. The etiology of bleeding included colonic diverticulosis (*n* = 16) and colonic cancer (*n* = 1). Bleeding was noted in the region of the right colonic artery (*n* = 8), ileocolic artery (*n* = 3), middle colonic artery (*n* = 2), and sigmoid artery (*n* = 4). The bleeding foci were in the ascending colon in 11 patients (65%), transverse colon in 2 patients (12%) and sigmoid colon in 4 patients (23%). Coagulopathy was defined as an international normalized ratio (INR) > 1.5 or a platelet count of < 8.0 × 10^4^/μL, and was observed in one patient.

**Table 1:** The background of the patients

Patient No.	Age(years)/sex	Cause of bleeding	Bleeding foci	Platelets (×10^4^/μL)	INR
1	58/M	Colonic diverticulosis	Ascending colon	15.9	1.44
2	66/M	Colonic diverticulosis	Ascending colon	18.0	1.24
3	79/M	Colonic diverticulosis	Ascending colon	30.1	1.14
4	81/M	Colonic diverticulosis	Sigmoid colon	14.7	1.35
5	72/M	Colonic diverticulosis	Ascending colon	27.5	1.09
6	69/M	Colonic diverticulosis	Ascending colon	18.3	NA
7	69/M	Colonic diverticulosis	Sigmoid colon	24.6	NA
8	63/M	Sigmoid colon cancer	Sigmoid colon	41.3	1.23
9	79/M	Colonic diverticulosis	Ascending colon	16.0	3.66
10	68/M	Colonic diverticulosis	Transverse colon	16.4	NA
11	54/M	Colonic diverticulosis	Ascending colon	24.4	1.18
12	74/M	Colonic diverticulosis	Transverse colon	14.0	1.11
13	52/M	Colonic diverticulosis	Ascending colon	15.8	NA
14	69/M	Colonic diverticulosis	Ascending colon	12.7	0.96
15	71/M	Colonic diverticulosis	Sigmoid colon	33.5	0.99
16	89/F	Colonic diverticulosis	Ascending colon	20.2	0.98
17	65/F	Colonic diverticulosis	Ascending colon	30.1	1.42

*M* male, F female, *INR* international normalized ratio, *NA* not applicable

The summary of TAE outcomes is shown in Table 2. Embolization was performed in the long branch (*n* = 10) and short branch (*n* = 8) of the vasa recta, with both long and short branches embolized in one case. Angiographic findings included extravasation (*n* = 16) and pseudoaneurysm (*n* = 1). The mean diameter of the embolized branch was 1.1 mm (range 0.5–1.5 mm). The mean follow-up period was 19 months (range 1–80 months).

**Table 2:** The summary of transcatheter arterial embolization outcomes

Patient No.	Embolized artery	Artery diameter (mm)	DSA finding	Combined length of all microcoils (cm)	Technical/Clinical success	Major complication	Early recurrent bleeding	Clinical follow-up (months)
1	RCA/LB of vasa recta	1.0	EV	4	Yes/Yes	None	None	80
2	RCA/LB of vasa recta	1.0	EV	4	Yes/Yes	None	None	44
3	ICA/SB of vasa recta	0.5	EV	2	Yes/Yes	None	None	16
4	SA/LB, SB of vasa recta	0.5, 0.7^a^	EV	16	Yes/Yes	None	None	11
5	ICA/LB of vasa recta	0.8	EV	4	Yes/Yes	None	None	10
6	RCA/LB of vasa recta	1.0	EV	4	Yes/Yes	None	None	8
7	SA/SB of vasa recta	1.2	PAN	4	Yes/Yes	None	None	48
8	SA/LB of vasa recta	1.4	EV	12	Yes/Yes	None	None	10
9	RCA/SB of vasa recta	1.2	EV	4	Yes/Yes	None	None	35
10	MCA/LB of vasa recta	1.2	EV	4	Yes/Yes	None	None	28
11	RCA/LB of vasa recta	0.7	EV	8	Yes/Yes	None	None	1
12	MCA/SB of vasa recta	1.0	EV	4	Yes/Yes	None	None	15
13	RCA/LB of vasa recta	1.5	EV	14	Yes/Yes	None	None	7
14	RCA/SB of vasa recta	1.0	EV	21	Yes/Yes	None	None	5
15	SA/SB of vasa recta	1.5	EV	4	Yes/Yes	None	None	1
16	ICA/LB of vasa recta	1.5	EV	12	Yes/Yes	None	None	1
17	RCA/SB of vasa recta	1.2	EV	9	Yes/Yes	None	None	1

*DSA* digital subtraction angiography, *RCA* right colic artery, *MCA* middle colic artery, *ICA* ileocolic artery, *SA* sigmoid artery, *LB* long branch, *SB* short branch, *EV* extravasation, *PAN* pseudoaneurysm

^a^Both long and short branches were embolized in this case (long branch, short branch)

Ultraselective TAE using small-sized microcoils was technically and clinically successful in all patients. Each embolization required two to eight small-sized microcoils (size range 1–3 mm; mean combined lengths of all microcoils 7.6 cm). There were no cases in which a microcoil migrated to the marginal artery, and none had early recurrent bleeding or major complications. Recurrent bleeding did occur in one patient at 2 months after TAE, but was stopped with conservative treatment.

## Discussion

Previous reports have shown recurrent bleeding rates after TAE of 10–28% and major complication rates of 5–19% (Hur et al. 2014; Kim et al. 2017; Kwon et al. 2019; Tan et al. 2010). In recent years, advances of microcatheter and microguidewire technologies have made it possible to perform pinpoint embolization for highly limited bleeding sites. This has made TAE for LGIB more effective and less invasive. Furthermore, superselective embolization (fewer than three embolized vasa recta) has been shown to be a significant prognostic factor associated with reduced recurrent bleeding and fewer major complications (Kwon et al. 2019).

The safety and efficacy of TAE with microcoils or NBCA for treatment of LGIB has been widely shown (d'Othée et al. 2006; Funaki et al. 2001; Huang et al. 2011; Hur et al. 2014; Kim et al. 2017; Kodani et al. 2016; Koganemaru et al. 2012; Kuo et al. 2003; Kwon et al. 2019; Park et al. 2020; Shimohira et al. 2015; Tan et al. 2010; Teng et al. 2013; Yata et al. 2013). Microcoils have several advantages, including their availability in various diameters, lengths, and shapes, and their visibility, which allows precise deployment. We were able to achieve accurate and ultraselective embolization in this study, with no non-target embolization due to coil migration. However, microcoils also have several disadvantages, including that the microcatheter must be advanced as far as possible to the bleeding site, which can lead to vasospasm and vessel injury (Vaidya et al. 2008). This also makes the procedure more difficult. However, no vasospasm or vessel injury occurred in the present study. Another disadvantage is that early recurrent bleeding is a concern, especially in patients with coagulation disorders.

The recurrent bleeding rate may be higher with microcoils than with NBCA embolization because of recanalization or collateral circulation due to proximal embolization (Hur et al. 2014; Kim et al. 2017; Kwon et al. 2019; Vaidya et al. 2008). Kwon et al. reported that use of NBCA was associated with lower rates of recurrent bleeding, although with no independent relationship with recurrent bleeding (Kwon et al. 2019). The use of NBCA carries risks of non-target embolization due to reflux and adherence of the microcatheter tip to the vessel wall. Thus, there is no consensus regarding use of embolic agents. However, NBCA may be useful in a setting of hemodynamic instability or coagulopathy (Hur et al. 2014; Kim et al. 2017; Kwon et al. 2019). In this study, we performed ultraselective TAE with microcoils for one patient with coagulopathy that became apparent after performing TAE. This patient had no recurrent bleeding. However, if coagulopathy had been apparent in advance, we may have considered use of NBCA.

In both the small intestine and colon, the vasa recta consists of long and short branches. There are rich anastomoses between the long branches, short branches, and both types of vessels (Kachlik et al. 2010). In a case of proximal embolization, the blood supply might be preserved by these anastomoses, including adjacent short and long branches. Koganemaru et al. showed the utility of ultraselective TAE with small-sized microcoils (Koganemaru et al. 2012). It is important to select a branch of the vasa recta with a thin-tipped microcatheter and to insert the microcatheter as close as possible to the bleeding site. This allows for short segment and dense embolization with small-sized microcoils at the level of branches of the vasa recta. This procedure may prevent recanalization and collateral circulation due to proximal embolization, which are disadvantages of embolization with microcoils, and reduce the risk of ischemia. Superselective TAE with microcoils in previous studies had rebleeding rates of 11–14% and major ischemic complication rates of 0–7% (Funaki et al. 2001; Kuo et al. 2003). In this study, there was no early recurrent bleeding or major complications. These results indicate that ultraselective TAE is a good option for LGIB. However, recurrent bleeding occurred in one patient 2 months after TAE. This patient had contrast extravasation at the same site found on a CT scan. Endoscopy confirmed hemostasis; hence, conservative treatment was performed. The patient had multiple diverticula at the same site and might have had bleeding from another diverticulum.

This study has several limitations. First, it was a retrospective feasibility study using a small sample size. Second, embolization was performed with microcoils only, and no comparison with other embolic agents was made. Third, colonoscopy after TAE was not performed routinely, and some ischemic complications might have been undetected in the absence of clinical symptoms. However, these complications can be classified as minor and they did not affect the clinical course of the patients. Fourth, bleeding foci in the small intestine and rectum were not included, and we did not assess small intestine or rectal bleeding. In particular, the rectum has a well-developed collateral circulation compared with the colon, and NBCA may be a useful embolic agent for rectal bleeding (Park et al. 2020). Thus, a prospective, randomized study comparing NBCA with microcoil embolization is required.

## Conclusion

Ultraselective TAE with small-sized microcoils is a highly effective and safe treatment modality for LGIB, and patients are unlikely to have early recurrent bleeding.

## Data Availability

The datasets used and/or analyzed during the current study are available from the corresponding author on reasonable request.

## Abbreviations

EMRs: Electronic medical records
IMA: Inferior mesenteric artery
LGIB: Lower gastrointestinal bleeding
NBCA: N-butyl cyanoacrylate
SMA: Superior mesenteric artery
TAE: Transcatheter arterial embolization

## References

[CR1] Barnert J, Messmann H (2009). Diagnosis and management of lower gastrointestinal bleeding. Nat Rev Gastroenterol Hepatol.

[CR2] d'Othée BJ, Surapaneni P, Rabkin D (2006). Microcoil embolization for acute lower gastrointestinal bleeding. Cardiovasc Intervent Radiol.

[CR3] Filippiadis DK, Binkert C, Pellerin O (2017). Cirse quality assurance document and standards for classification of complications: the cirse classification system. Cardiovasc Intervent Radiol.

[CR4] Funaki B, Kostelic JK, Lorenz J (2001). Superselective microcoil embolization of colonic hemorrhage. Am J Roentgenol.

[CR5] Huang CC, Lee CW, Hsiao JK (2011). N-butyl cyanoacrylate embolisation as the primary treatment of acute hemodynamically unstable lower gastrointestinal hemorrhage. J Vasc Interv Radiol.

[CR6] Hur S, Jae HJ, Lee M (2014). Safety and efficacy of transcatheter arterial embolisation for lower gastrointestinal bleeding: a single-center experience with 112 patients. J Vasc Interv Radiol.

[CR7] Kachlik D, Baca V, Stingl J (2010). The spatial arrangement of the human large intestinal wall blood circulation. J Anat.

[CR8] Kim PH, Tsauo J, Shin JH (2017). Transcatheter arterial embolization of gastrointestinal bleeding with n-butyl cyanoacrylate: a systematic review and meta-analysis of safety and efficacy. J Vasc Interv Radiol.

[CR9] Kodani M, Yata S, Ohuchi Y (2016). Safety and risk of superselective transcatheter arterial embolization for acute lower gastrointestinal hemorrhage with n-butyl cyanoacrylate: angiographic and colonoscopic evaluation. J Vasc Interv Radiol.

[CR10] Koganemaru M, Abe T, Iwamoto R (2012). Ultraselective arterial embolization of vasa recta using 1.7-French microcatheter with small-sized detachable coils in acute colonic hemorrhage after failed endoscopic treatment. Am J Roentgenol.

[CR11] Kuo WT, Lee DE, Saad WE (2003). Superselective microcoil embolization for the treatment of lower gastrointestinal hemorrhage. J Vasc Interv Radiol.

[CR12] Kwon JH, Kim M-D, Han K (2019). Transcatheter arterial embolization for acute lower gastrointestinal haemorrhage: a single-centre study. Eur J Radiol.

[CR13] Park S, Kim Y, Shin JH (2020). Outcome of rectal arterial embolization for rectal bleeding in 34 patients: a single-center retrospective study over 20 years. J Vasc Interv Radiol.

[CR14] Shimohira M, Hashizume T, Ohta K (2015). Triaxial transarterial embolization for lower gastrointestinal bleeding: a retrospective case series. Minim Invasive Ther Allied Technol.

[CR15] Tan KK, Nallathamby V, Wong D (2010). Can superselective embolization be definitive for colonic diverticular hemorrhage? An institution’s experience over 9 years. J Gastrointest Surg.

[CR16] Teng HC, Liang HL, Lin YH (2013). The efficacy and long-term outcome of microcoil embolotherapy for acute lower gastrointestinal bleeding. Korean J Radiol.

[CR17] Vaidya S, Tozer KR, Chen J (2008). An overview of embolic agents. Semin Interv Radiol.

[CR18] Yata S, Ihaya T, Kaminou T (2013). Transcatheter arterial embolization of acute arterial bleeding in the upper and lower gastrointestinal tract with n-butyl-2-cyanoacrylate. J Vasc Interv Radiol.

